# Lithium-Ion Migration-Induced Magnetic Anisotropy Transition in CoNi Thin Films

**DOI:** 10.3390/ma19143012

**Published:** 2026-07-13

**Authors:** Zhen Han, Senmiao Liu, Ronghuan Xie, Ziwen Meng, Yawen Li, Qiang Cao

**Affiliations:** 1 Spintronics Institute, University of Jinan, Jinan 250022, China; 202321100167@stu.ujn.edu.cn (Z.H.); 202311100002@stu.ujn.edu.cn (S.L.); 15953683953@139.com (Z.M.); liyw9@stu.ujn.edu.cn (Y.L.); 2 State Key Laboratory of Crystal Materials, School of Physics, Shandong University, Jinan 250100, China; xrhh1124@sdu.edu.cn

**Keywords:** voltage-controlled magnetic anisotropy, lithium-ion batteries, interfacial electric field, CoNi films

## Abstract

**Highlights:**

- Applying a voltage sweep from 3.0 V to 1.5 V reorients the CoNi magnetic anisotropy from perpendicular toward the in-plane direction.
- The magnetic anisotropy transition is driven by Li^+^ ion accumulation in TiO_2_, rather than intercalation into CoNi.
- Consecutive charge–discharge cycles indicate that the magnetic anisotropy modulations are reversible and repeatable.

**Abstract:**

Voltage control of magnetic anisotropy in nanoscale heterostructures plays a pivotal role in the design and realization of magnetic random-access memories. To achieve low-voltage operation with high reversibility, we introduce an ion-conducting TiO_2_ layer capable of storing lithium ions atop a Ta/Pt/CoNi heterostructure. During the discharging process, lithium ions migrate into the TiO_2_ layer. The resulting interfacial electric field between TiO_2_ and CoNi induces a reversible evolution of magnetic anisotropy from the out-of-plane direction toward the in-plane direction. Within a voltage window of 1.5 V (from 3.0 V to 1.5 V), both remanent magnetization and coercivity are suppressed to zero. Furthermore, consecutive charge–discharge cycles indicate the reversibility in the modulation of remanent magnetization and coercivity. These findings highlight that ion migration at the magnetic interface enables efficient and reversible control of magnetic anisotropy, opening new opportunities for the development of low-power spintronic devices.

## 1. Introduction

Conventional current-driven magnetic switching, such as spin-transfer torque or spin–orbit torque, still faces major challenges due to high energy consumption, Joule heating, and scalability limitations [1,2,3,4,5]. By comparison, voltage-driven control offers a fundamentally more efficient pathway, making it particularly attractive for next-generation non-volatile memories [6,7]. Voltage control of magnetic anisotropy (VCMA) can be achieved through four main mechanisms: dielectric gating, piezostrain, multiferroic coupling, and magneto-ionic effects [8,9]. Each mechanism exploits different physical principles to modulate magnetic anisotropy using electric fields, offering distinct advantages and limitations for spintronic applications [10,11]. Dielectric gating uses electric fields to modulate charge density at ferromagnet/oxide interfaces, offering fast response and compatibility with tunnel junctions, but the effect size is relatively small and limited to ultrathin films [12,13,14]. Piezostrain control relies on voltage-induced strain in piezoelectric or ferroelectric substrates to alter magnetoelastic energy, enabling strong and reversible anisotropy changes, though it requires high voltage and can be nondurable [15,16]. Multiferroic coupling exploits the intrinsic interaction between ferroelectric and ferromagnetic orders, allowing compact multifunctional devices, but suitable materials with strong coupling at room temperature are scarce [17,18]. Finally, the magneto-ionic effect involves voltage-driven ion intercalation or redox reaction, producing deeper and larger changes in magnetic properties. However, the main limitations of magneto-ionic control are in terms of reversibility and stability [19,20,21].

Such limitations are tied to the fundamental nature of ion migration. The ions may become trapped in unintended sites, diffuse too deeply, or cause local structural changes that are not easily undone, leading to incomplete recovery of the original magnetic state over repeated cycles [22,23]. Stability is also a concern, as redistributed ions can drift or relax even after the voltage is removed, especially under thermal stress, gradually degrading the controlled anisotropy. In addition, repeated ion motion may introduce defects or chemical inhomogeneity, reducing endurance. Thus, while magneto-ionic control offers robust switching advantages, ensuring fully reversible ion migration and long-term stability remains the key challenge for practical device applications [24,25,26].

In this work, we demonstrate the reversible and reliable voltage control of magnetic anisotropy in Ta/Pt/CoNi/TiO_2_ heterojunctions enabled by lithium-ion migration. Previous studies [6] demonstrated modulation of Ruderman–Kittel–Kasuya–Yosida (RKKY) coupling in Co/Ni-based antiferromagnets via oxygen ion migration. However, this redox-driven mechanism induced slow dynamics and degraded the reversible cycling performance. In our device architecture, TiO_2_ was deliberately selected as the ion-conducting layer because of its ion-storage capability and high ionic diffusion coefficient. The ions are primarily stored within the TiO_2_ layer, preventing deep bulk intercalation into the CoNi magnetic layer and thereby minimizing structural degradation during operation. Instead, the interfacial electric field generated at the CoNi/TiO_2_ interface drives the magnetic anisotropy transition, significantly enhancing both reversibility and reliability. Applying a voltage sweep from 3.0 V to 1.5 V reorients the magnetic anisotropy of CoNi from the perpendicular direction toward the in-plane direction. Moreover, consecutive charge–discharge cycles indicate good reversibility in the manipulation of magnetic anisotropy. These results highlight the effectiveness of interfacial ion migration in achieving stable voltage-controlled magnetic anisotropy and underscore the potential of this approach for the development of reliable low-power spintronic devices.

## 2. Materials and Methods

The Ta (2 nm)/Pt (2 nm)/CoNi (0.4 nm)/TiO_2_ (3 nm) heterostructure was deposited on thermally oxidized Si (001) substrates by magnetron sputtering at room temperature. All depositions were performed under an Ar atmosphere with a pressure of 3 mTorr. The deposition rates were calibrated by X-ray reflectivity (XRR) measurements [27]. Prior to the sputtering process, the chamber pressure was maintained below 5 × 10^−8^ Torr. Pt and TiO_2_ targets were sputtered in radio frequency (RF) mode, while Co, Ni, and Ta targets were deposited in direct current (DC) mode. Lithium-ion batteries were assembled in an argon-filled glove box, using the as-grown heterostructure as the cathode and lithium metal as the counter electrode. The electrolyte consisted of 1.0 M LiPF_6_ dissolved in a 1:1 w/w mixture of dimethyl carbonate (DEC) and ethylene carbonate (EC). A Celgard 2250 film (Celgard, Charlotte, NC, USA) was used as the separator. The gate voltage was applied using a Keithley 2450 (Tektronix, Cleveland, OH, USA), while the current was supplied by a Keithley 6221 (Tektronix, Cleveland, OH, USA) and the voltage was recorded with a Keithley 2182 (Tektronix, Cleveland, OH, USA). All the measurements were conducted at room temperature. Magnetic loops were characterized using a Quantum Design Magnetic Property Measurement System (MPMS, Quantum Design, San Diego, CA, USA).

## 3. Results and Discussion

**Figure 1:**
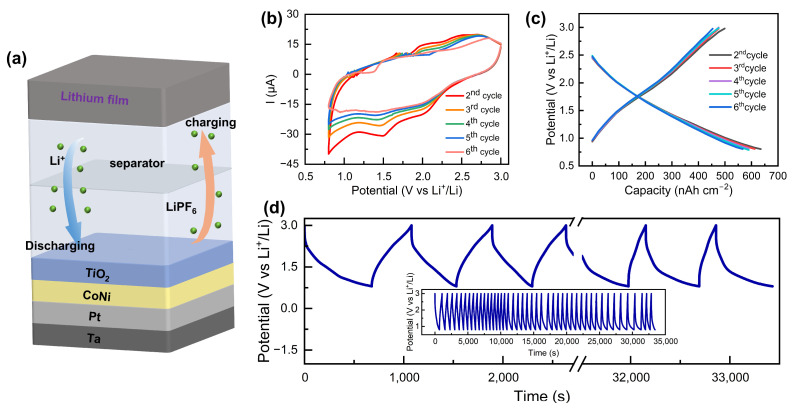
(**a**) Schematic illustration of the device structure and voltage-induced charging and discharging behavior. (**b**) The voltammogram curves from the second to sixth cycles between 3.0 V and 0.8 V at a scan rate of 10 mV·s^−1^. (**c**) Galvanostatic charge–discharge profiles corresponding to the CV at a constant current of 10 µA. (**d**) Galvanostatic charge–discharge measurements recorded over 45 cycles with potential from 3 to 0.8 V.

Figure 1a schematically illustrates the device structure based on lithium-ion migration. During discharging, Li^+^ ions migrate through the electrolyte and separator and accumulate in the TiO_2_ layer. During charging, the Li^+^ ions are extracted from the TiO_2_ layer and migrate back to the lithium electrode. The cyclic voltammogram (CV) curves are shown in Figure 1b. The differences in the first six cycles are due to the formation of the solid electrolyte interphase (SEI) and/or structural activation [28]. With increasing cycling, the curves tend to overlap, evolving toward pseudocapacitive characteristics [29]. The galvanostatic charge–discharge curves in Figure 1c exhibit quasi-linear slopes with the absence of distinct voltage plateaus, which is also consistent with the pseudocapacitive behavior. The cycling stability was evaluated through galvanostatic charge–discharge measurements, as shown in Figure 1d. During repeated application of voltages between 0.8 V and 3.0 V, our device indicates good reversibility for over 45 cycles.

**Figure 2:**
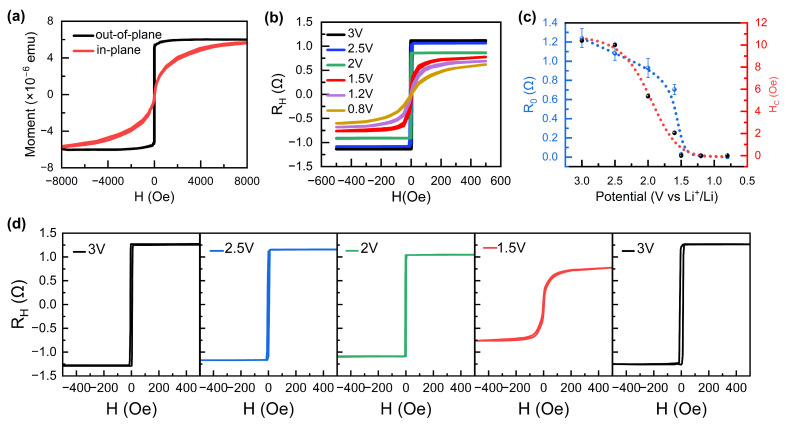
Voltage-controlled magnetic properties of the CoNi/TiO_2_ film. (**a**) In-plane and out-of-plane magnetization hysteresis loops measured in the pristine state. (**b**) The Hall resistance loops under various voltages from 3 V to 0.8 V. To achieve a stable state, each voltage is maintained for 10 min before measurements. (**c**) Dependence of remanent Hall resistance and coercivity on the applied voltage, varied from 3.0 V to 0.8 V. Error bars represent the standard deviation of five independent measurements. (**d**) Hall resistance loops measured during a voltage sweep from 3.0 to 1.5 V and back to 3 V.

The magnetic properties of a Ta/Pt/CoNi/TiO_2_ multilayer are shown in Figure 2a. The saturation magnetization of CoNi film was measured to be 1.08 × 10^6^ A m^−1^. The variation in the magnetic anisotropy energy, $∆K_{i}$, was evaluated from hard-axis magnetization measurements. The value of $∆K_{i}$ was determined using the relations

(1) $$∆K_{i}=1/2∆H_{k}\mu_{0}M_{S}d_{CoNi}$$

(2) $$∆H_{K}=H_{K,IP}+H_{K,P}$$

where $d_{CoNi}$ = 0.4 nm. From the measurements in Figure 2a, with $H_{K,IP}$ ~ 7000 Oe and $H_{K,P}$ ~ 20 Oe, the change in magnetic anisotropy energy is estimated to be $∆K_{i}$ ~ 0.15 mJ m^−2^. To provide a comparison with other systems, a preliminary estimate of the magnetoelectric coupling coefficient *α*_ME_ is provided. Using ∆V = 1.5 V, $\alpha_{ME}$ is calculated to be on the order of 100 fJ V^−1^ m^−1^ [30]. Figure 2b presents the Hall loops under various voltages. Before each Hall resistance measurement, the voltage is applied and held for 10 min, in order to reach a steady state. The loop retains a square shape in the voltage range from 3.0 to 2.0 V, indicating a stable out-of-plane magnetic anisotropy. When the voltage is further reduced to 1.5 V, the magnetic easy axis switches towards the in-plane direction. It is maintained as the voltage continues to decrease down to 0.8 V. The dependence of remanent Hall resistance (*R*_0_) and coercivity (*H*_C_) on different voltages from 3.0 V to 0.8 V is shown in Figure 2c. In the as-grown state (3.0 V), *R*_0_ is approximately 1.25 Ω, and *H*_C_ is about 10.5 Oe. Upon discharging, both *R*_0_ and *H*_C_ decrease sharply. At 1.5 V, they approach zero, indicating a transition of the magnetic easy axis from the perpendicular towards the in-plane direction. The successive evolution of Hall loops during voltage sweeps from 3.0 V to 1.5 V and back to 3 V is shown in Figure 2d. As the voltage decreases from 3.0 V to 1.5 V, magnetic anisotropy reorients from the perpendicular direction towards the in-plane direction. Upon recharging to 3.0 V, magnetic anisotropy is fully recovered to the out-of-plane direction [31].

**Figure 3:**
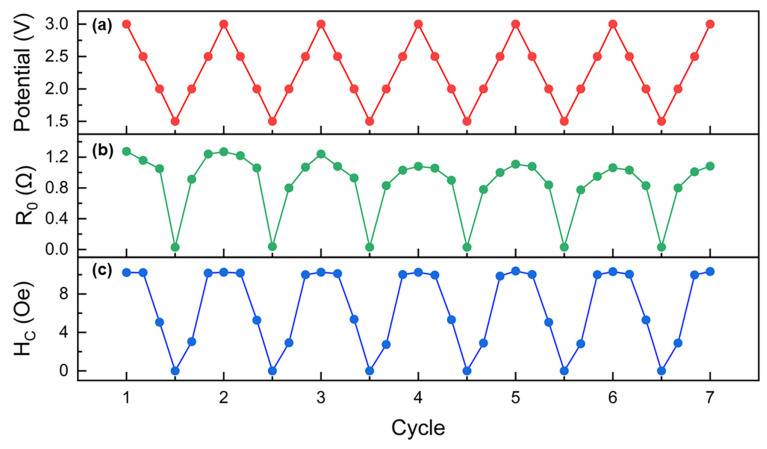
Reversible voltage control of magnetic anisotropy in CoNi films. (**a**) The applied voltage repeated between 3.0 and 1.5 V in steps of 0.5 V. At each voltage, the system was held for 10 min prior to measurement to ensure a steady state. (**b**) Dependence of *R*_0_ on the applied voltage. (**c**) Dependence of *H*_C_ on the applied voltage. The data points in (**b**,**c**) represent single sequential measurements recorded at each voltage step to trace the continuous cyclic evolution.

Reversible and reliable magnetic anisotropy modulations are demonstrated in CoNi films over six cycles. As shown in Figure 3a, the applied voltage is stepped from 3.0 V to 1.5 V in increments of 0.5 V. The evolution of the *R*_0_ and *H*_C_ over six consecutive voltage cycles is presented in Figure 3b and Figure 3c, respectively. The six cycles are shown here due to the long time required for voltage stabilization and magnetic measurements. Those cycles are representative of the steady-state device operation. Our results reveal that the magnetic modulations can be observed if the device can work, which is more than 45 cycles. Both the remanent Hall signal and coercivity exhibit a clear and reproducible dependence on the applied voltage, indicating a strong coupling between the electric field and magnetic properties. In particular, repeatable switching between perpendicular and in-plane anisotropy is observed across consecutive cycles, indicating good reversibility of the modulations. In addition, the in-plane magnetic state gradually relaxes back towards the initial perpendicular state without a power supply. This volatile behavior further corroborates our proposed interfacial electric field mechanism. Once the accumulated Li^+^ ions diffuse back, the external electric field is removed, and then magnetic anisotropy recovers.

This work demonstrates a strategy for achieving reversible voltage-controlled magnetic anisotropy in a Ta/Pt/CoNi/TiO_2_ heterostructure. Here, TiO_2_ serves as an ionic conductor, while the CoNi metal layer suppresses Li ion insertion. Therefore, we believe that Li ions migrate into the TiO_2_ layer and generate a strong interfacial electric field at the CoNi/TiO_2_ interface during discharging, thereby modulating the magnetic anisotropy. This electric field modifies the occupation of the 3d orbitals in the CoNi layer and consequently alters magnetic anisotropy. In addition, the electric field enables us to change the interfacial Rashba interaction arising from inversion symmetry breaking, which contributes to the anisotropy variation.

## 4. Conclusions

In conclusion, we demonstrate VCMA in Ta/Pt/CoNi/TiO_2_ heterostructures mediated by interfacial lithium-ion accumulation. By largely confining Li^+^ ions within the TiO_2_ layer and suppressing extensive intercalation into the CoNi layer, structural degradation is minimized and reversible operation is maintained. Therefore, the voltage-driven interfacial electric field enables a controllable switching of magnetic anisotropy from the perpendicular direction toward the in-plane direction, with good reversibility over the evaluated cycles. These findings provide an effective strategy for achieving robust voltage-controlled magnetism and offer promising prospects for the development of low-power, high-reliability spintronic devices. Nevertheless, the present system represents a reversible model platform rather than a device-ready switching scheme. Achieving practical, high-speed, non-volatile memory operation will require further optimization, particularly in fully solid-state architectures and device integration.

## Data Availability

The original contributions presented in this study are included in the article. Further inquiries can be directed to the corresponding author.

## Abbreviations

The following abbreviations are used in this manuscript:

CV	Cyclic Voltammogram
MRAM	Magnetoresistive Random Access Memory
MPMS	Magnetic Property Measurement System
VCMA	Voltage-Controlled Magnetic Anisotropy
*R*_0_	Remanent Hall resistance
*H*_C_	Coercivity
